# A novel *in silico *reverse-transcriptomics-based identification and blood-based validation of a panel of sub-type specific biomarkers in lung cancer

**DOI:** 10.1186/1471-2164-14-S6-S5

**Published:** 2013-10-25

**Authors:** Debmalya Barh, Neha Jain, Sandeep Tiwari, John K Field, Elena Padin-Iruegas, Alvaro Ruibal, Rafael López, Michel Herranz, Antaripa Bhattacharya, Lucky Juneja, Cedric Viero, Artur Silva, Anderson Miyoshi, Anil Kumar, Kenneth Blum, Vasco Azevedo, Preetam Ghosh, Triantafillos Liloglou

**Affiliations:** 1 Centre for Genomics and Applied Gene Technology, Institute of Integrative Omics and Applied Biotechnology (IIOAB), Nonakuri, Purba Medinipur, WB-721172, India; 2 School of Biotechnology, Devi Ahilya University, Khandwa Road Campus, Indore, MP, India; 3 University of Liverpool, Department of Molecular and Clinical Cancer Medicine, 200 London Road, Liverpool L3 9TA, UK; 4 Medical Oncology Department, Complejo Hospitalario Universitario, Santiago de Compostela, A Coruña, Spain; 5 Nuclear Medicine Service, Complejo Hospitalario Universitario. Fundación Tejerina. Santiago de Compostela, A Coruña, Spain; 6 Molecular Oncology and Imaging Program, Complejo Hospitalario Universitario, Santiago de Compostela, A Coruña, Spain; 7 Institute of Molecular and Experimental Medicine, Cardiff University, Cardiff CF14 4XN, Wales, UK; 8 Instituto de Ciências Biológicas, Universidade Federal do Pará, Rua Augusto Corrêa, 01 - Guamá, Belém, PA, Brazil; 9 Laboratorio de Genetica Celular e Molecular, Departmento de Biologia Geral, Instituto de Ciencias Biologics, Universidade Federal de Minas Gerais CP 486, CEP 31270-901 Belo Horizonte, Minas Gerais, Brazil; 10 Department of Psychiatry and Mcknight Brain Institute, College of Medicine, University of Florida, University Ave., Gainesville, FL 32601, USA; 11 Department of Computer Science and Center for the Study of Biological Complexity, Virginia Commonwealth University, Richmond, Virginia, USA

## Abstract

Lung cancer accounts for the highest number of cancer-related deaths worldwide. Early diagnosis significantly increases the disease-free survival rate and a large amount of effort has been expended in screening trials and the development of early molecular diagnostics. However, a gold standard diagnostic strategy is not yet available. Here, based on miRNA expression profile in lung cancer and using a novel *in silico *reverse-transcriptomics approach, followed by analysis of the interactome; we have identified potential transcription factor (TF) markers that would facilitate diagnosis of subtype specific lung cancer. A subset of seven TF markers has been used in a microarray screen and was then validated by blood-based qPCR using stage-II and IV non-small cell lung carcinomas (NSCLC). Our results suggest that overexpression of HMGA1, E2F6, IRF1, and TFDP1 and downregulation or no expression of SUV39H1, RBL1, and HNRPD in blood is suitable for diagnosis of lung adenocarcinoma and squamous cell carcinoma sub-types of NSCLC. Here, E2F6 was, for the first time, found to be upregulated in NSCLC blood samples. The miRNA-TF-miRNA interaction based molecular mechanisms of these seven markers in NSCLC revealed that HMGA1 and TFDP1 play vital roles in lung cancer tumorigenesis. The strategy developed in this work is applicable to any other cancer or disease and can assist in the identification of potential biomarkers.

## Introduction

Lung cancer is the leading cause among cancer related deaths worldwide, constituting 17% of new cancer cases and 23% of deaths from cancer. Although N. American and European countries show a slow decline in death rates due to lung cancer, deaths due to this form of cancer are increasing considerably in Asian and African countries [1]. Lung cancer is mainly divided into two subtypes, small cell lung cancer (SCLC), which accounts for 10-15% of all cases and non-small cell lung cancer (NSCLC, 85-90%). The latter group is further histologically subdivided into four categories; adenocarcinoma, squamous cell carcinoma, large cell carcinoma and 'others', for example cancers of neuroendocrine origin [2]. The overall 5-year survival rate for NSCLC ranges from 9% to 15% [3]. The high mortality from lung cancer is due a combination of lack of reliable early diagnostic tools [3,4] along with a poor arsenal of lung cancer regimens for stage I lung cancer, whose survival rate is also surprisingly low [5].

Numerous studies have utilized different "-omics"-based approaches to identify molecular signatures in lung cancer with diagnostic or prognostic value while using minimally invasive processes. Some of these are as follows: 34 miRNA signatures [6], expression profiles of 11 miRNAs (miR-106a, miR-15b, miR-27b, miR-142-3p, miR-26b, miR-182, miR-126, let7g, let-7i and miR-30e-5p) from serum [7], 7 miRNA signatures [8], overexpression of six snoRNAs [9], and expression of 3 miRs (miR-205, miR-210 and miR-708) in sputum [10]. Additional signatures and markers have also been reported from the plasma proteome [11,12], the salivary proteome [13], the serum epigenome [14], sputum-based genomics [15], and blood-based gene expression studies [16]. However, none of these have progressed sufficiently to provide the necessary specificity and sensitivity required for clinical implementation.

microRNAs (miRNAs/miRs) are involved in a variety of biological processes, including cell cycle regulation, cell differentiation, development, metabolism, and aging [17]. They have also been shown to be aberrantly expressed in several cancers [18]. Lung cancer is no exception to this and miRNA signatures have been suggested to be useful in diagnosis, prognosis, and therapy [7,19-21]. miRNAs regulate posttranscriptional gene expression and a single miRNA can regulate up to 200 mRNAs including those for transcription factors (TFs) [22]. Because miRNA transcription is under the regulation of TFs, intriguing feed-back and feed-forward regulatory loops can be formed among TFs and miRNAs [17].

In this study we have developed a novel *in silico *reverse-transcriptomics strategy followed by interactome analysis to identify the sub-type specific diagnostic TF markers in lung cancer. The approach is novel as the sub-type specific TF markers were identified starting with experimentally validated miRNA profiles in lung cancer. We have also attempted to provide a molecular insight during the early events in lung cancer.

## Materials and methods

### Literature mining

Extensive literature and text mining was carried out to collect deregulated miRNAs in lung cancers (NSCLC and SCLC) using databases such as PubMed, Sirus, and Elsevier as well as search engines such as Google and Google Scholar. miR2Disease [23] was also used to gather lung cancer specific miRNAs information. Priority was given to reports that have used markers based on biopsy samples and patient's remote media (blood, serum, plasma, sputum, and bronchioalveolar lavage among others [24]). Selected miRNAs were then grouped into three categories: (1) NSCLC specific, (2) exclusively SCLC related, and (3) common in both the types. The up- and down-regulated miRNAs within each of these three groups were also noted.

### GO assignment to miRNAs using reverse annotation strategy

**Figure 1:**
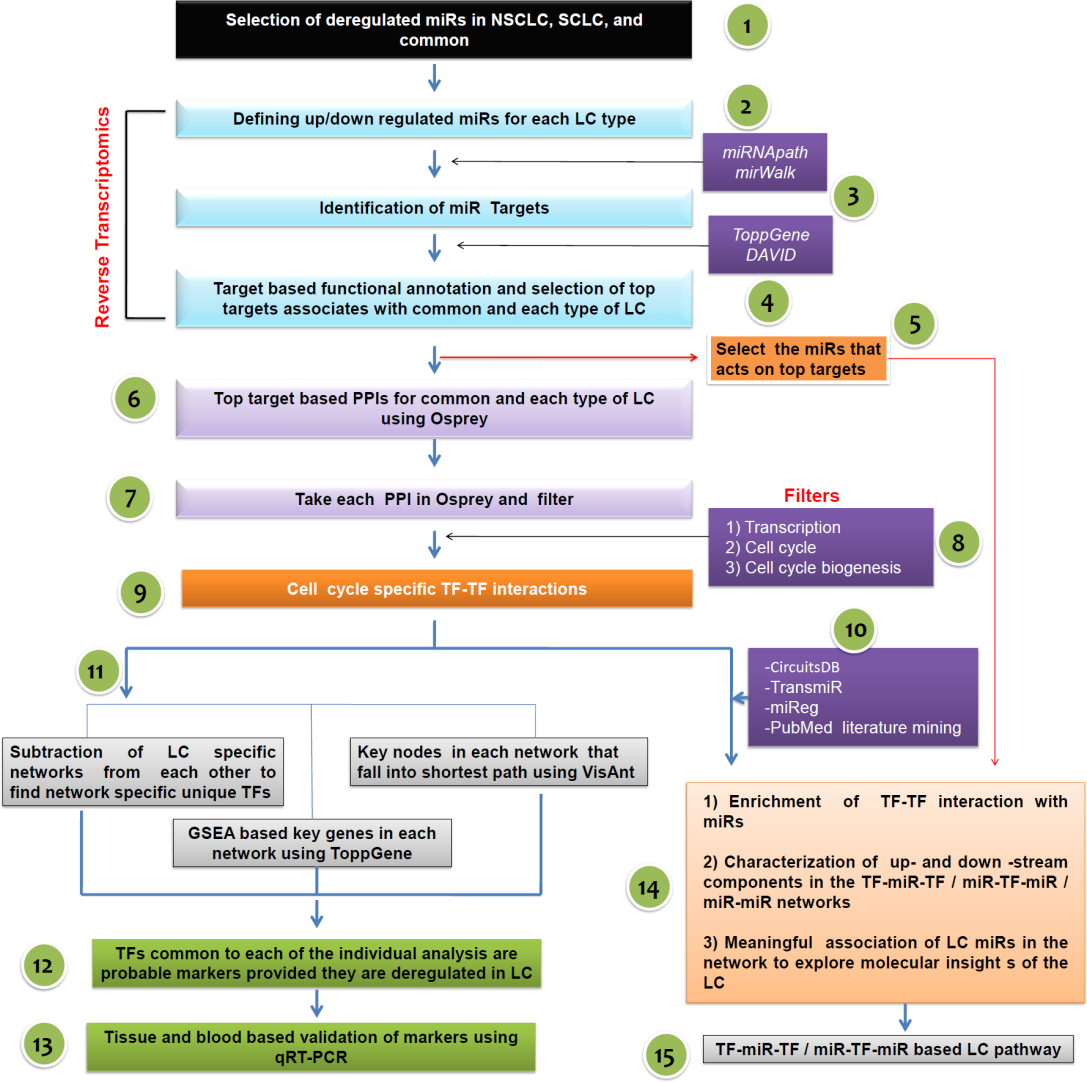
**Flow-diagram showing entire strategy that is applied to identify TF biomarkers in Lung cancer based on miRNA profiles**.

No tool is currently available to classify or cluster miRNAs as per their GO (Gene Ontology) or functional annotation. We applied a reverse approach in which GO terms to a miRNA are assigned based on the functional annotation of the targets of the particular miRNA. In this approach, we first identified experimentally validated targets of each miRNA using miRNA target databases miRWalk [25], miRecords [26], miReg [17], and miRTarBase [27]. Next, targets for each miRNA were subjected to ToppGene Suite [28] for GSEA (Gene Set Enrichment Analysis) candidate gene prioritization. The top-ranked genes were used in DAVID v6.7 [29] analysis for functional annotation clustering and the assignment of GO terms to each miRNA which targets these genes. GO terms related to various aspects of cancer were considered. miRNAs and their corresponding targets that fall under these specific GO categories were selected, and the rest were ignored (Figure 1, Step-3).

### miRNA-TF-miRNA or TF-miRNA-TF interactions

To date, there is no study reporting direct miRNA-miRNA interaction. However, it is well known that miRNAs can modulate post-transcriptional gene regulation as well as their own expression through feed-back and feed-forward loops that are mediated by various TFs. Therefore, there are miRNA-TF interactions. As TFs interact with other TFs and proteins, the known TF-TF networks can be complemented by integrating the relevant miRNA-TF interactions to make TF-miRNA-TF or TF-miRNA-TF-miRNA interactions. Such TF-miRNA-TF-miRNA interaction networks will indirectly represent the miRNA-miRNA interactions.

We thus created a cancer specific TF-TF interaction network using targets of miRNAs frequently deregulated in NSCLC, SCLC, or common to both of these types utilizing Osprey v1.0.1 [30] (Figure 1, Step-3). To achieve this, we selected all experimentally validated, highly ranked miRNA targets of NSCLC, SCLC, or common to both that were identified in the previous step and fed them into Osprey (Figure 1, Step-6). The protein-protein interaction (PPI) network for each cancer type generated by Osprey was first filtered sequentially with the "Transcription", "Cell cycle" and "Cell cycle biogenesis" GO filters in Osprey (Figure 1, Step-8). Therefore, the resultant TF-TF interaction network is cell cycle specific. The sequential filters were used because cell cycle deregulation is one of the major BPs (Biological Processes) that is affected during tumorigenesis.

This cell cycle specific TF-TF network was further enriched by manually mapping the interacting miRNAs with data collected from the miReg [17], TransmiR [31], and CircuitsDB [32] databases and from literature mining to create a TF-miRNA-TF interaction map (Figure 1, Step-10). Because we have selected lung cancer related miRNAs (based on GO assignment in the previous step) and developed a network using their targets, this network represents the interaction of TFs involved in lung cancer tumorigenesis. Based on our earlier hypothesis, this interaction map also represents the miRNA-TF-miRNA or TF-miRNA-TF interaction map that is common to both NSCLC and SCLC. Similarly, NSCLC and SCLC specific miRNA-TF-miRNA or TF-miRNA-TF or miRNA-miRNA interaction maps were created using targets of NSCLC and SCLC unique miRNAs. Therefore, a total of three networks were generated (Figure 1, Steps-14-15).

### Marker identification

The miRNA-TF-miRNA or TF-miRNA-TF interaction maps for NSCLC, SCLC, and common developed in the previous steps were analyzed by subtracting from each other to identify the NSCLC, SCLC, and a common pathway that is specific unique TFs. Each network was further analyzed using the protein-protein interaction (PPI) analysis tool VisANT [33] to identify the key nodes and the shortest cancer specific pathways in each network. Key nodes in a PPI network are identified as having the highest number of interactions. Therefore, such key node proteins are often involved in multiple signaling pathways, and if a key node protein falls in a shortest path, the node might be treated as a marker of a disease provided that its expression is altered in that disease state. In the third strategy, we utilized GSEA identification of key genes in each network using ToppGene Suite [28]. When all of the data from each of these three analyses had been obtained, we identified the TFs common to each of the individual analyses (Figure 1, Steps-11-12). Therefore, these sets of common TFs were putative markers, and the TFs that were a part of NSCLC network could be treated as a NSCLC-specific marker.

### Experimental validation of markers

Once we had selected the potential markers, we checked their expression levels initially in lung cancer tissue samples using microarrays and then further validated them using patient's blood samples and quantitative RT-PCR (qPCR) (Figure 1, Step-13).

#### Interrogation of data from expression microarray

The frozen tissue samples examined from 30 squamous cell carcinomas and 30 adenocarcinomas (each is a type of NSCLC) from the Liverpool Lung Project tissue bank. All samples were of pathological stage T2. RNA was extracted using the RNeasy kit (Qiagen). Five RNA pools from five adjacent normal lung tissues were also profiled for comparison purposes. The microarray experiments were performed by Almac (Belfast, UK). Total RNA was amplified using the NuGEN™ Ovation™ RNA Amplification System V2. First-strand synthesis of cDNA was performed using a unique first-strand DNA/RNA chimeric primer mix, resulting in cDNA/mRNA hybrid molecules. Following fragmentation of the mRNA component of the cDNA/mRNA molecules, second-strand synthesis was performed, and double-stranded cDNA was produced with a unique DNA/RNA heteroduplex at one end. In the final amplification step, RNA within the heteroduplex was degraded using RNaseH, and a replication of the resultant single-stranded cDNA was achieved using the DNA/RNA chimeric primer binding and DNA polymerase enzymatic activity. The amplified single-stranded cDNA was purified to allow accurate quantitation of the cDNA and to ensure optimal performance during the fragmentation and labeling process. The single-stranded cDNA was assessed using spectrophotometric methods in combination with the Agilent Bioanalyzer.

The appropriate amount of amplified single-stranded cDNA was fragmented and labeled using the FL-Ovation™ cDNA Biotin Module V2. The enzymatically and chemically fragmented product (50-100 nt) was labeled via the attachment of biotinylated nucleotides onto the 3'-end of the fragmented cDNA.

The resultant fragmented and labeled cDNA was added to the hybridization cocktail in accordance with the NuGEN™ guidelines for hybridization onto Affymetrix GeneChip^® ^arrays. Following hybridization for 16-18 hours at 45°C in an Affymetrix GeneChip^® ^Hybridization Oven 640, the array was washed and stained on the GeneChip^® ^Fluidics Station 450 using the appropriate fluidics script and then inserted into the Affymetrix autoloader carousel and scanned using the GeneChip^® ^Scanner 3000.

The Rosetta Error Model has been applied to the raw data to generate the processed data. The profile comparisons between cancerous lesions and normal RNA pools utilized Student's t-test. The Benjamini & Hochberg multiple test correction method was also employed.

#### Validation using quantitative RT-PCR (qPCR)

##### Blood samples, RNA isolation, and cDNA preparation

As our focus is NSCLC, blood samples from 8 metastatic lung adenocarcinoma, 8 metastatic squamous cell lung carcinoma patients, and 5 healthy volunteers (control) were used for the validation. Patient eligibility criteria were as follows: 18 years of age or older, in clinical stage II-IV based on the International TNM classification, performance status of 0 to 2, and no other malignances. All patients and volunteers have signed informed consent forms. Ten milliliters of EDTA blood sample was collected from the selected groups before chemotherapy treatment. Blood samples were centrifuged at 2000 g for 10 min and the serum phase was separated and frozen at -80ºC. The Buffy Coat (white blood cells and circulating tumor cells) was collected and processed by lysis (Ammonium Chloride, TRIS, ddH_2_0) and then washed with PBS. The dry pellet was kept at -80ºC until RNA isolation. RNA was purified by Quiamp RNA Blood Mini Kit (QIAGEN Inc., USA) according to the manufacturer´s instructions. cDNA was synthesized with random hexamer primers (Deoxynucleoside Triphosphate set, Roche, Germany) at 10 mM, MgCl_2_, MuLV Reverse Transcriptase, PCR Buffer, RNAse Inhibitor, and random hexamers from Applied Biosystems USA. The resulting cDNA was stored at -20ºC until further use.

#### Quantitative RT-PCR (qPCR)

qPCR was carried out using SYBR^® ^Green Master Mix (Applied Byosistems, USA) and Applied Biosystem's 7500 real-time PCR system according to the manufacturer´s instructions. Primers for GAPDH were designed with Vector NTI Advance™ 11 (Invitrogen) and primers for TFDP1, SUV39H1, RBL1, E2FG, IRF1, HMGA1, and HNRPD were designed using *qPrimerDepot *(http://primerdepot.nci.nih.gov/). To avoid the influence of genomic contamination, the amplicons spanned at least one intron. The primers used are listed in Additional file 1. qPCR was performed in a final volume of 20 µl with a SYBR PCR Master Mix, using 1 µl cDNA. Cycling conditions were 95ºC for 10 min, followed by 40 cycles at 95ºC for 15 s and 60ºC for 1 min each to obtain the melting curve.

Relative gene expression levels were determined by the quantitative curve method. Quantitative normalization of the cDNA in each sample was performed using GAPDH gene expression as an internal control. Target gene mRNA levels were given as ratios to GAPDH mRNA levels. qPCR assays were performed in duplicate for each sample, and the mean value was used to calculate the mRNA expression levels.

## Results

### miRNA statistics in lung cancer

We selected 184 miRNAs for NSCLC and 62 for SCLC using literature mining and the miR2 Disease database. Among these 246 miRNAs, 41 were found to be involved in both of the lung cancers and therefore are common miRNAs involved in lung cancer regardless of the subtype (Figure 1, Step-1). In the common miRNA group, 13 and 11 miRNAs were found to be up- and downregulated, respectively; whereas 18 miRNAs showed differential expression, i.e., either upregulated in SCLC and downregulated in NSCLC or *vice versa *(Figure 1, Step-2) (Additional file 2). A total of 22 miRNAs were found to be unique to SCLC (16 upregulated and 6 downregulated) (Additional file 3). For NSCLC, the total number of unique miRNAs was 143, (89 upregulated and 43 downregulated) (Additional file 4).

### Target-based functional annotation of miRNAs

Using miRWalK, miRBASE, miRecord, miRTarBASE, and miReg we identified several validated targets for each miRNA. Thereafter, as per our reverse transcriptomics strategy, targets for each miRNA were subjected to gene enrichment analysis using ToppGene Suite as described in Materials and Methods (Figure 1, Step-3). Top targets that are associated with common, NSCLC, and SCLC were identified. DAVID-based functional annotations of the top targets revealed that most of these targets are cell cycle related, so the miRNAs that have these targets are related to transcription, cell cycle regulation, cell biogenesis and organization, cell proliferation, and other biological processes related to tumorigenesis. The list of common miRNAs involved in lung cancer along with their corresponding GO terms is presented in Additional file 5. miRNAs involved uniquely in either NSCLC or SCLC and their corresponding GO terms were also defined (data not shown).

### miRNA-miRNA interaction network in lung cancer

#### Interaction of common miRNAs

**Figure 2:**
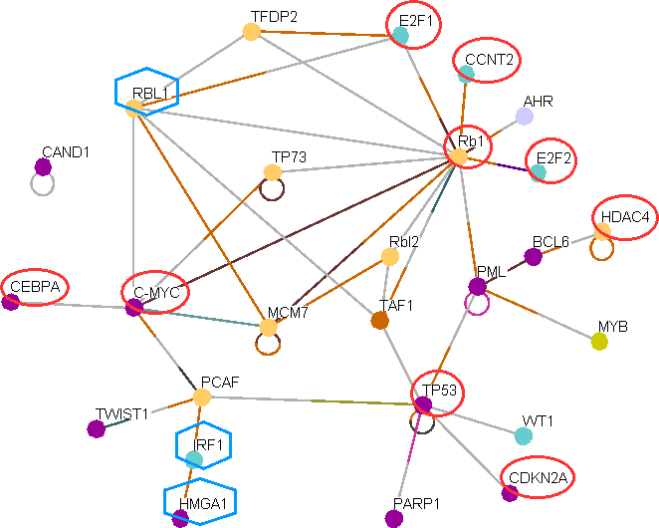
**Cell cycle specific 26 key interacting TFs that are targets of miRNAs involved in common events in lung cancer as well as in NSCLC and SCLC**. The network is created as described in the text. As per our hypothesis, this network also represents interactions of cell cycle regulating miRNAs associated with NSCLC, SCLC, and common events of lung cancer. TFs circled in red are shared by both NSCLC and SCLC. Molecules marked in hexagon are unique to common events. Other molecules in the map are shared by NSCLC and common events of lung cancers.

Based on the hypothesis that interactions of miRNA-TF-miRNA or TF-miRNA-TF-miRNA targets represent miRNA-miRNA interactions, we used gene enrichment based on the top targets of miRNAs common to NSCLC and SCLC in Osprey to create a protein-protein interaction map (Figure 1, Steps-6-7). In total, 638 targets corresponding to 40 common miRNAs generated a map having 1791 nodes in Osprey. Keeping in mind that miRNA genes are regulated by transcription factors (TF), miRNAs regulate TFs, and, as the gene enrichment analysis shows, most of the miRNAs regulate transcription, the network of 1791 nodes is filtered with the "Transcription factor" filter in Osprey and subsequently only 170 nodes are retained. This transcription network of 170 nodes is further filtered with "Cell cycle" and "Cell Organization and Biogenesis" filters, as per the enriched GO categories (Figure 1, Step-8), and finally the cell cycle specific total of 26 key TF nodes in common events, NSCLC, and SCLC are found (Figure 1, Step-9 and Figure 2).

#### Interactions of SCLC associated miRNAs

**Figure 3:**
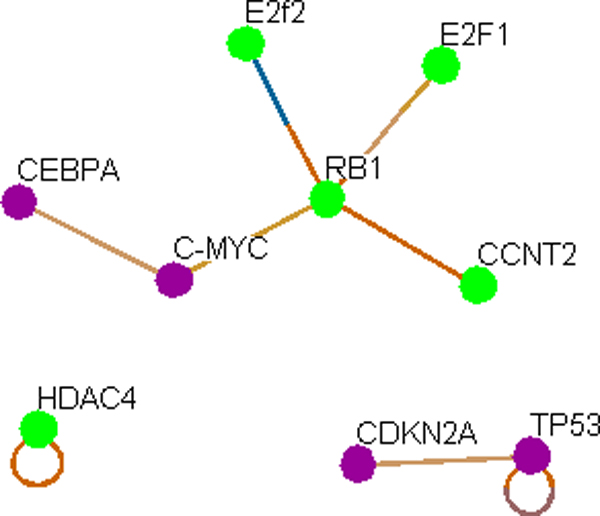
**Cell cycle specific 9 key interacting TFs that are targets of miRNAs involved in SCLC**. As per our hypothesis, this network represents interaction of cell cycle regulating miRNAs associated with SCLC. For detail, please see the text.

For SCLC, 634 nodes are used in total to create the interaction map in Osprey. The resultant map is sequentially filtered with "transcription factor", "Cell cycle", and "Cell organization and biogenesis" Filters and only 9 key nodes are obtained (Figure 1, Steps-6-9 and Figure 3).

#### Interactions of NSCLC linked miRNAs

**Figure 4:**
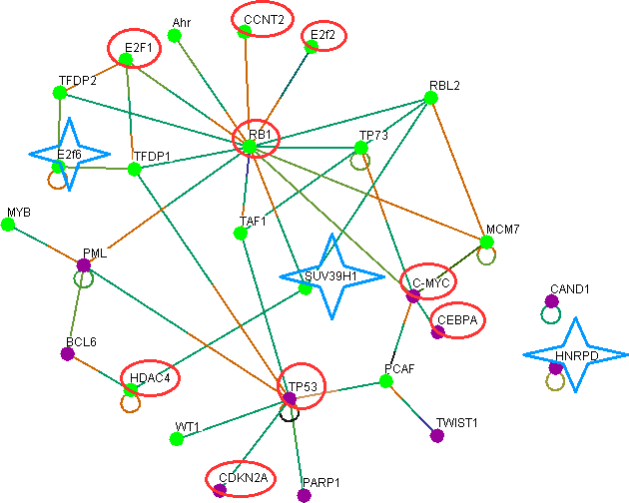
**Interactions of TFs (as per our hypothesis miRNAs) associated with NSCLC and SCLC**. TFs circled in red are shared by both NSCLC and SCLC. Molecules marked in star are unique to NSCLC. Other molecules in the map are shared by NSCLC and general events of lung cancers.

Similar methods of network creation and filtering to those applied to identify key nodes in common and in SCLC (Figure 1, Steps-6-9) were adopted to generate a key interaction network in NSCLC. A total of 2421 nodes are filtered and finally 27 nodes are obtained (Figure 4).

#### SCLC network is a part of NSCLC

Next we subtracted the LC specific networks from each other to identify unique network specific TFs (Figure 1, Step-11). In the 27 nodes of the NSCLC network (Figure 4), all of the SCLC nodes (Figure 2) are found to be present (Figure 4, in red circle). Therefore, it is evident that there are additional pathways involved in NSCLC compared to SCLC and the SCLC network represents a subset of the NSCLC network.

### Genes involved in common events in lung cancer

Next, we compared the common network (Figure 2) with the SCLC (Figure 3) and NSCLC and SCLC networks (Figure 4) by subtracting each from the other to identify key nodes that are common to (1) SCLC and NSCLC; (2) general events, NSCLC, and SCLC; (3) NSCLC and general; (4) NSCLC specific; and (5) general events in lung cancers. The analysis revealed that nine genes (RB1, E2F1, E2F2, CCNT2, CMYC, CEBPA, TP53, CDKN2A, and HDAC4) that are key nodes in SCLC are common to both the (1) SCLC and NSCLC and (2) general events, NSCLC, and SCLC groups (Table 1, group-1-3). Therefore, all of the SCLC genes are involved in NSCLC and in general events in lung cancer. Fourteen unique genes (Table 1, group-4) are found to be involved in both NSCLC and general events. The comparison also shows that four genes (Table 1, group-5) are specific to NSCLC and three genes (Table 1, group-6) are unique to general events. Therefore, these gene sets can be used in combination and their expression signature may be useful as diagnostic markers for NSCLC.

**Table 1:** Identified putative markers in lung cancers using the *in silico *reverse transcriptomics approach.

Group	LC Types	Gene sets
1	Unique to SCLC	RB1, E2F1, E2F2, CCNT2, CMYC, CEBPA, TP53, CDKN2A, HDAC4
2	Common to SCLC and NSCLC	RB1, E2F1, E2F2, CCNT2, CMYC, CEBPA, TP53, CDKN2A, HDAC4
3	Common to general, SCLC, and NSCLC	RB1, E2F1, E2F2, CCNT2, CMYC, CEBPA, TP53, CDKN2A, HDAC4
4	Common to NSCLC and general	TFDP2, AHR, CCND1, TP73, RBL2, TAF1, PML, BCL6, MYB, WT1, PARP1, PCAF, TWIST, MCM7
5	NSCLC specific	E2F6, TFDP1, SUV39H1, HNRPD
6	General/ common path specific	RBL1, IRF1, HMGA1

The markers can be used in combination to design panels for diagnosis of sub-type specific lung cancers.

### Validation of markers

We selected seven genes [4 unique genes (E2F6, TFDP1, SUV39H1, and HNRPD) for NSCLC and 3 genes (RBL1, IRF1, and HMGA1) for general events] for validation as diagnostic markers in lung cancer. Frozen NSCLC tissue-based microarray analysis revealed that E2F6, TFDP1, SUV39H1, and HMGA1 are significantly upregulated in both the adenocarcinoma and squamous cell carcinoma samples. The upregulation of RBL1 and downregulation of IRF1 in the microarray analysis was significant in squamous cell carcinoma but was statistically insignificant in adenocarcinoma (Additional file 6).

**Figure 5:**
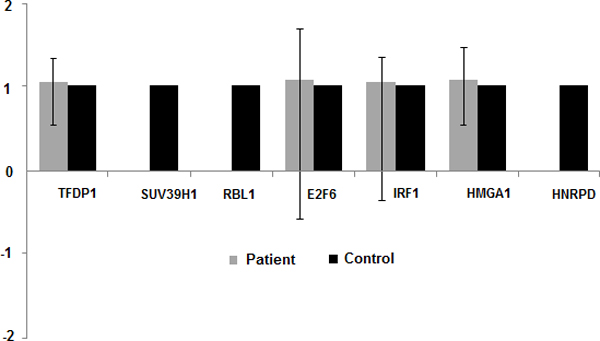
**Blood based qPCR results for selected seven NSCLC specific markers**. As compared to the control; HMGA1, TFPD1, E2F6, and IRF1 are upregulated and SUV39H1, RBL1, and HNRPD are downregulated or not expressed in all tested samples.

qPCR validation of markers based on blood samples showed expression patterns similar to the tissue based microarray analysis. TFPD1, E2F6, IRF1, and HMGA1 are upregulated in all cancer samples. SUV39H1, RBL1, and HNRPD are downregulated or not expressed in all samples compared to the control (Figure 5). Therefore, combining the microarray and qPCR results, upregulation of E2F6, HMGA1, IRF1, and TFDP1 and downregulation or no expression of SUV39H1, RBL1, HNRPD can be used as diagnostic markers of NSCLC, and, in particular, adenocarcinoma and squamous cell carcinoma.

## Discussion

In this work we have identified key transcription factors that can be useful biomarkers in diagnosis of lung cancer using an *in silico *reverse-transcriptomics approach. In this novel approach, starting with deregulated miRNAs in lung cancers we have identified transcription factors that can act as biomarkers, even for sub-type specific lung cancers. Out of several putative markers we identified, 7 NSCLC specific markers were validated. We found that E2F6, HMGA1, IRF1, and TFDP1 were upregulated and RBL1, SUV39H1, and HNRPD were downregulated or aberrantly expressed in adenocarcinoma and squamous cell carcinoma, which are the sub-types of NSCLC.

**HMGA1 **(High mobility group AT-hook 1) is an oncogene that is induced by Wnt/beta-catenin pathway and which positively regulates cell proliferation in gastric cancer [34]. By downregulating E-cadherin and upregulating expression of TWIST1, it enhances epithelial-mesenchymal transition and metastasis in colon cancer [35]. Upregulation of HMGA1 in glioblastoma positively correlates with malignancy, angiogenesis, and invasion [36]. In lung cancer, it is also overexpressed and increased nuclear expression correlates with poor survival in lung adenocarcinomas [37,38]. By upregulating PI3K and MMP2, it promotes cell migration and invasion [37,39] and by activating miR-222 oncomiR, it induces PPP2R2A mediated AKT signaling in NSCLC [40]. Therefore, upregulation of HMGA1 plays a significant role in tumor progression in NSCLC. In our study, we also observed that HMGA1 was upregulated in NSCLC supporting the previous findings.

**TFDP1 **(Transcription factor Dp-1) is a candidate oncogene that positively regulates S-phase entry and inhibits apoptosis in cooperation with E2F1 [41]. It is amplified and overexpressed in breast cancer [42] and upregulation of TFDP1 positively correlates with tumor size and progression of hepatocellular carcinomas [43] and increased cell viability in lung cancer [44]. In our observation, TFDP1 was overexpressed in all lung adenocarcinomas and squamous cell carcinomas, which supports the previous findings of Lu et al. (2000) in a SCLC cell line [45].

In our study, we observed **IRF1 **(Interferon regulatory factor 1) was upregulated in all NSCLC samples tested, although it had been shown to be downregulated in lung cancer in a previous study [46]. IRF1 inhibits G1-S cell cycle progression through P53 and p21 mediated pathways [46] and may act as a tumor-suppressor gene. This finding is supported by the findings that it is downregulated in gastric [47] and recurrent breast cancers [48]. However, IRF1 may not always act as a tumor-suppressor, as there is a report that it is upregulated in skin squamous cell carcinoma [49]. Therefore, our observation of upregulated IRF1 in NSCLC samples requires further attention to explore the precise role of this TF in various cancers.

**E2F6 **(E2F transcription factor 6) inhibits entry into S phase of cells stimulated to exit G0 [50] and inhibits apoptosis through E2F1 [51]. It may therefore play a role in cell proliferation and cell survival. There is no report about this protein's expression pattern in any cancer. Here, we have, for the first time, observed that E2F6 was upregulated in all of our tested NSCLC samples. This finding supports E2F6's putative role in tumorigenesis and shows that it may be a novel marker for NSCLC.

**SUV39H1 **(Suppressor of variegation 3-9 homolog 1) is a histone methyltransferase that inhibits inflammatory responses by downregulating interleukin-6 production [52]. SUV39H1 inhibits the expression of CCND1 and may thereby negatively regulate cell proliferation [53]. However, its overexpression induces cell migration in breast and colon cancers [54] and negatively regulates apoptosis in a lung cancer model [55]. The expression level of SUV39H1 inversely correlates with stage, prognosis, and disease free survival in oral squamous cell carcinoma [56] and breast cancer [57]. Therefore, SUV39H1 may also have oncogenic properties. Although SUV39H1 was significantly upregulated in adenocarcinoma and squamous cell carcinoma tissue samples in our microarray analysis, supporting its positive role in tumorigenesis, it was found to be downregulated in blood samples in our qPCR validation. Therefore, SUV39H1 expression differs in lung cancer tissue and blood samples.

**RBL1 **(Retinoblastoma-like 1 (p107)) inhibits cell proliferation through G1 arrest [58] and positively regulates epidermal differentiation [59]. RBL1 is downregulated and inversely correlates with the histological grade of squamous cell carcinomas and adenocarcinomas [60]. Our qPCR validation shows downregulation in all squamous cell carcinoma and adenocarcinoma samples, which supports the previous findings and RBL1's function in tumors.

**HNRPD/AUF1 **is a RNA-binding protein that both positively and negatively regulates neoplastic gene regulatory networks in cancer depending on the type of neoplasm [61]. It binds to destabilize p21 mRNA and thereby inhibits its anti-apoptotic activity [62]. Although in our blood-based qPCR analysis AUF1 was downregulated in all NSCLC samples, it has been reported to be upregulated in HCC [63] and experimental murine lung cancer [64]. It has been patented to aid in the prediction of survival in lung cancer in a gene expression panel of biomarkers (US 20100267574).

**Figure 6:**
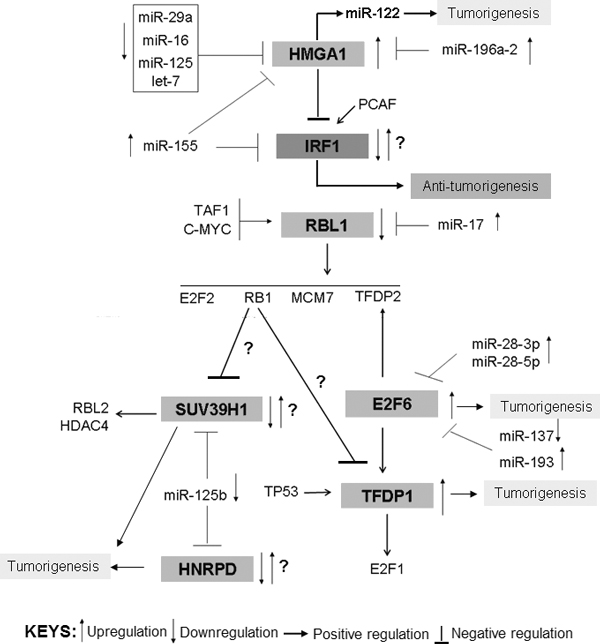
**The correlations of identified seven TF markers and interacting miRNAs**. The interactions provide better insights of molecular events and mechanisms during lung cancer tumorigenesis. For detail, please see the text.

**miRNA-markerTFs correlation**: The seven identified TFs that are aberrantly expressed in both the squamous cell carcinoma and adenocarcinoma were plotted for their interactions with miRNAs and other key TFs to obtain more insight into these markers in lung cancer pathogenesis (Figure 1, Steps-14-15). The miRNA-TF-Cancer relationships were gathered from the miReg [17], miR2Disease [23], miRWalk [25], miRecords [26], TransmiR [31], CircuitsDB [32], and miRDB [65] databases. The interaction map is represented in Figure 6.

The network clearly shows meaningful relationships between the TFs and miRNAs in lung cancer. The interactions show that the tumor suppressor miRNAs (miR-29a, miR-16, miR-125, and let-7) that could target the oncogene HMGA1 are downregulated. Upregulation of HMGA1 induces expression of oncogenic miR-122. Another two pro-oncogenic miRNAs that can also target HMGA1, miR-196a-2 and miR-155, are upregulated in lung cancers [66,67]. We observed that HMGA1 may inhibit the putative tumor-suppressor IRF1 (as per the interaction network) and that the miR-155 pro-oncomiR directly targeted IRF1. Therefore, in this network, HMGA1 is the key TF that positively regulates lung tumorigenesis through upregulation of miR-122 and perhaps by downregulation of IRF1. However, we found that IRF1 is upregulated in the samples so that the IRF1-HMGA1 interactions need further attention.

Tumor suppressor RBL1 is a target of the miR-17 oncomiR [68]. Furthermore, as per the interaction network, RBL1 is activated by TAF1 and cMYC, and regulates expression of E2F2, RB1, MCM7, and TFDP2. It thereby regulates the cell cycle and cell proliferation. Therefore, RBL1 downregulation and upregulation of miR-17 provide a meaningful mechanism in lung cancer tumorigenesis [66,69].

The common pathway (of both NSCLC and SCLC) related genes HNRPD, E2F6, TFDP1, and SUV39H1 also showed the expected TF-miRNA relationship in the interaction map represented in Figure 6 based on the available experimental evidence. The literature shows that HNRPD and SUV39H1 may have positive roles in tumorigenesis [55,56,64]. Although in our blood-based qPCR, HNRPD and SUV39H1 are downregulated, they are reported to be upregulated in a mouse model of lung cancer [63], consistent with the tissue-based microarray analysis in our lung cancer samples. The involvement of HNRPD and SUV39H1 is further supported by reports that the tumor suppressor miR-125 is downregulated in both NSCLC and SCLC [70,71]. Furthermore, the tumor suppressor protein RB1 is downregulated in lung cancer [66] and may inhibit SUV39H1.

The other two markers, E2F6 and TFDP1, are upregulated in all of our blood samples. While two pro-oncogenic miRNAs, miR-28 and miR-193, are upregulated [40] the putative tumor-suppressor, miR-137, is downregulated in lung cancers [72,73]. All three of these miRNAs target E2F6 [74,75]. Furthermore, E2F6 putatively upregulates TFDP1 and is downregulated by RB1. It is also found from the interaction map that E2F6 inhibition by two upregulated pro-oncomiRs (miR-28 and miR-193) is not sufficient, as the E2F6 was found to be upregulated in lung cancer. Further, E2F6 has been reported to upregulate oncogene TFDP1 and to positively regulate cell proliferation and cell survival through E2F1 [41]. Additionally, downregulation of RB1 in lung cancer is not able to repress TFDP1 activity, and therefore, in lung cancer, tumorigenesis is mediated through upregulation of E2F6 and TFDP1. However, the role of SUV39H1 and HNRPD requires further exploration.

## Conclusion

In this analysis, using an integrated reverse-transcriptomics-based bioinformatics approach, we have identified key transcription factors that may be useful in developing subtype specific biomarkers in lung cancer. Our proposed seven markers also have high potential to be used in lung cancer diagnostics for NSCLC subtypes. Of course, additional experimental validation in independent sets of patients is required to establish the diagnostic accuracy of this panel and we are currently conducting those experiments. The miRNA-TF-miRNA relationships with these seven miRNAs show meaningful associations with these TFs in lung cancer pathogenesis. The novel strategy developed in this research is powerful and can be applicable to identify molecular mechanisms and markers in other cancers as well.

## Funding

This work was carried out without any grant. VA had funding from CNPq and FAPEMIG.

## Conflict of interest

Authors declare no conflict of interest.

## Authors' contributions

DB: Conceived the idea, designed the study, coordinated and leaded the entire project, and wrote the manuscript; **DB, NJ: **collected and analyzed primary data, **DB, NJ, ST, AB, LJ: **performed all *in silico *analyses; **JKF, TL: **performed microarray analysis; **EP, AR, RL, MH: **performed qPCR experiments; **PG**, **CV**, **AK, AS, AM, VA, KB: **cross verified all analyses. All authors have read and approved the manuscript.

## Supplementary Material

Additional file 1 **List of primers to amplify TFDP1, SUV39H1, RBL1, E2FG, IRF1, HMGA1, and HNRPD**. Click here for file

Additional file 2 **Common miRNAs involved in both NSCLC and SCLC**. The differentially expressed miRNAs are marked with blue. Click here for file

Additional file 3 **Small-cell-lung cancer (SCLC) specific 22 deregulated miRNAs (16 upregulated and 6 downregulated)**. Click here for file

Additional file 4 **Non-small-cell lung cancer (NSCLC) specific 143 deregulated miRNAs (89 upregulated and 43 downregulated)**. The miRNAs that are reported upregulated in one report but downregulated in other report or *vise versa *are highlighted in blue. Click here for file

Additional file 5 Functional annotation of common miRNAs using the targets of these miRNAs and DAVID Click here for file

Additional file 6 **Microarray based expression analysis of NSCLC specific 6 identified markers [E2F6, TFDP1, and SUV39H1 for NSCLC and RBL1, IRF1, and HMGA1 for general events]**. E2F6, TFDP1, SUV39H1, and HMGA1 are significantly upregulated in both the adenocarcinoma and squamous cell carcinoma samples. The upregulation of RBL1 and downregulation of IRF1 in the microarray analysis was significant in squamous cell carcinoma but was statistically insignificant in adenocarcinoma. Click here for file

## Declarations

Publication for this article has been funded by NSF 1158608.

This article has been published as part of *BMC Genomics *Volume 14 Supplement 6, 2013: Proceedings of the International Conference of the Brazilian Association for Bioinformatics and Computational Biology (X-meeting 2012). The full contents of the supplement are available online at http://www.biomedcentral.com/bmcgenomics/supplements/14/S6.
