# Supplementary material for: A novel in silico reverse-transcriptomics-based identification and blood-based validation of a panel of sub-type specific biomarkers in lung cancer
Source: BMC Genomics. 2013 Oct 25;14(Suppl 6):S5. doi: 10.1186/1471-2164-14-S6-S5 (PMC3908344; doi:10.1186/1471-2164-14-S6-S5)
Supplement: Additional file 1 — List of primers to amplify TFDP1, SUV39H1, RBL1, E2FG, IRF1, HMGA1, and HNRPD. [file 1471-2164-14-S6-S5-S1.doc]

**Additional file –1:** List of primers to amplify TFDP1, SUV39H1, RBL1, E2FG, IRF1, HMGA1, and HNRPD.

| **Marker Genes** | **Primers** |
| --- | --- |
| HMGA1 | Forward: 5'-CCCCGAGGTCTCTTAGGTGT-3'  Reverse: 5'-AAAAGGACGGCACTGAGAAG-3' |
| HNRPD | Forward: 5'-TCCCAGCTAAGGCCTCCTAT-3'  Reverse: 5'-ACGCCAGTAAGAACGAGGAG-3' |
| TFDP1 | Forward: 5'-CTTTCCCGGGACTAAGGTTC-3'  Reverse: 5'-TTTCCCGGATCTGGTAACAT-3' |
| SUV39H1 | Forward: 5'-ACGTCCTCCACGTAGTCCAG-3'  Reverse: 5'-ATTCGCAAGAACAGCTTCGT-3' |
| RBL1 | Forward: 5'-TGACATGTCCATCCATTTCT-3'  Reverse: 5'-AAGCATTATTCCCACGGTTG-3' |
| E2F6 | Forward: 5'-CCAGCGATACATCAAAACGA-3'  Reverse: 5'-AGTTACCCAGTCTCCTCCTG-3' |
| IRF1 | Forward: 5'-ATCCTTGTTGATGTCCCAGC-3'  Reverse: 5'-GACCCTGGCTAGAGATGCAG-3' |
