# Supplementary material for: A novel in silico reverse-transcriptomics-based identification and blood-based validation of a panel of sub-type specific biomarkers in lung cancer
Source: BMC Genomics. 2013 Oct 25;14(Suppl 6):S5. doi: 10.1186/1471-2164-14-S6-S5 (PMC3908344; doi:10.1186/1471-2164-14-S6-S5)
Supplement: Additional file 2 — Common miRNAs involved in both NSCLC and SCLC. The differentially expressed miRNAs are marked with blue. [file 1471-2164-14-S6-S5-S2.doc]

**Additional file -2:** Common miRNAs involved in both NSCLC and SCLC. The differentially expressed miRNAs are marked with blue.

| **miRNA** | **UP/DOWN in Small Cell lung cancer** | **References for small lung cancer** | **UP/DOWN in Non-small Cell lung Cancer** | **Refrences for non small cell lung cancer** |
| --- | --- | --- | --- | --- |
| mir-9 | Up | PMID: 19895320 | DOWN | PMID: 16530703 |
| miR-15a | UP | PMC: 2907339 | DOWN | PMID: 18766170 |
| miR-16 | UP | PMC: 2907339 | DOWN | PMID: 18766170 |
| miR-17-5p, | UP | PMC: 2907339, PMID: 16266980, PMC: 2907339 | UP | PMID: 16461460 |
| miR-20a, | UP | PMID: 16266980 | UP | PMID: 20818338 |
| miR-22 | DOWN | PMC: 2907339 | DOWN | PMID: 18766170 |
| miR-23a | DOWN | PMC: 2907339 | UP in PMID: 18766170 | PMID: 18766170, NSCLC (Korean paper) |
| miR-23b | DOWN | PMC: 2907339 | UP | PMID: 18766170 |
| miR-25 | Up | PMID: 19895320 | UP | PMID: 18766170 |
| miR-24 | DOWN | PMC: 2907339 | DOWN | PMID: 18766170 |
| miR-27a | DOWN | PMC: 2907339 | UP in PMID: 18766170 | NSCLC (Korean paper) |
| miR-29 | Down | PMID: 19895320 | DOWN | PMID: 17890317 |
| miR-29a | DOWN | PMC: 2907339 | DOWN | ,PMID: 17890317 |
| miR-29b | DOWN | PMC: 2907339 | DOWN | PMID: 17890317 |
| miR-29c | DOWN | PMC: 2907339 | DOWN | PMID: 17890317, NSCLC |
| miR-34a | Down | PMID: 19895320 | UP | PMID: 16461460 |
| miR- 92a-2 | UP | PMID: 20548249 | DOWN | PMID: 18766170 |
| miR-93 | UP | PMC: 2907339 | UP | PMID: 19584273 |
| miR-95 | Up | PMID: 19895320 | DOWN | PMID: 16530703, NSCLC (Korean paper) |
| miR-99a | DOWN | PMC: 2907339 | UP | PMID: 18766170, NSCLC (Korean paper) |
| miR-101 | UP | PMC: 2907339 | DOWN | PMID: 18766170, NSCLC (Korean paper) |
| miR-103 | UP | PMC: 2907339 | UP | PMID: 18766170 |
| miR-106a cluster | Up | PMID: 19895320 | UP | PMID: 19584273 |
| miR-106b | UP | PMID: 16266980 ,PMC: 2907339 | UP | PMID: 19584273 |
| miR-107 | UP | PMC: 2907339 | UP | PMID: 18766170 |
| mir-126 | Down | PMID: 19895320 | DOWN | PMID: 18602365, PMID: 21116241 , PMID: 19493678, PMID: 18766170, PMID: 20198613, NSCLC (Korean paper), PMID: 16461460 |
| miR-128b | Up | PMID: 19895320 | UP | PMID: 18766170, PMID: 16461460 |
| miR-135 | UP | PMC: ID: PMC: 2907339 | DOWN | PMC: ID: PMC: 2907339 |
| miR-142-3p | UP | PMC: 2907339 | DOWN | PMID: 19228723 |
| miR-143 | Down | PMID: 19895320 | DOWN | PMID: 19228723, PMID: 18766170, |
| miR-145 | Down | PMID: 19895320 | DOWN | PMID: 19228723, PMID: 19493678, PMID: 21289483 PMID: 18766170, PMID: 16530703, PMID: 20198613 |
| miR-149 | UP | PMC: 2907339 | UP | PMID: 16461460 |
| miR-150 | Down | PMID: 19895320 | UP | PMID: 18766170 |
| miR-155 | Down | PMID: 19895320 | UP | PMID: 16530703, PMID: 16461460 |
| mir-183 | Up | PMID: 19895320 | UP | PMID: 19493678, |
| miR-221 | DOWN | PMC: 2907339 | UP | PMID: 19962668 , PMID: 18766170, PMID: 18167339 |
| mmiR-222 | DOWN | PMC: 2907339, PMID: 19895320 | UP | PMID: 19962668 ,PMID: 18766170 |
| miR-205 | DOWN | PMC: 2907339 | UP | PMID: 18719201, PMID: 20526284, PMID: 19273703, PMID: 18766170, PMID: 16461460 |
| miR-223 | Down | PMID: 19895320 | UP | PMID: 18766170 |
| miR-224 | Down | PMID: 19895320 | DOWN | PMID: 16530703, PMID: 18766170 |
| miR-328 | UP | PMC: 2907339 | UP | PMID: 20818338, PMID: 18766170 |
