# Supplementary material for: A novel in silico reverse-transcriptomics-based identification and blood-based validation of a panel of sub-type specific biomarkers in lung cancer
Source: BMC Genomics. 2013 Oct 25;14(Suppl 6):S5. doi: 10.1186/1471-2164-14-S6-S5 (PMC3908344; doi:10.1186/1471-2164-14-S6-S5)
Supplement: Additional file 3 — Small-cell-lung cancer (SCLC) specific 22 deregulated miRNAs (16 upregulated and 6 downregulated). [file 1471-2164-14-S6-S5-S3.doc]

**Additional file -3:** Small-cell-lung cancer (SCLC) specific 22 deregulated miRNAs (16 upregulated and 6 downregulated).

| **miRNA** | **Lung Cancer Type** | **Sample** | **Up/Down regulation** | **References** |
| --- | --- | --- | --- | --- |
| miR-17-92 cluster | SCLC | Cell lines and tissue samples | Up | PMID: 16266980 |
| miR-17-3p | SCLC |  | Up | PMID: 16266980 |
| miR-15b | SCLC | Cell lines | Up | PMC: 2907339 |
| miR-18a | SCLC | Cell lines and tissue samples | Up | PMID: 16266980 |
| miR-19a | SCLC | Cell lines and tissue samples | Up | PMID: 16266980 |
| miR-19b | SCLC | Cell lines and tissue samples | Up | PMID: 16266980 |
| miR-31 | SCLC | Cell lines | Down | PMC: 2907339 |
| miR-33 | SCLC |  | Down | PMID: 19895320 |
| miR-92 | SCLC | Cell lines | Up | PMC: 2907339 |
| miR-92–1 | SCLC | Cell lines and tissue samples | Up | PMID: 16266980 |
| miR-96 | SCLC | Cell lines | Up | PMC: 2907339 |
| miR-98 | SCLC | Cell lines | Up | PMC: 2907339 |
| miR-125 | SCLC |  | Down | PMID: 19895320 |
| miR-148b | SCLC | Cell lines | Up | PMC: 2907339 |
| miR-199a | SCLC | Cell lines | Down | PMC: 2907339 |
| miR-200c | SCLC |  | Up | PMID: 19895320 |
| miR-301 | SCLC |  | Up | PMID: 19895320,PMC: 2907339 |
| miR-338 | SCLC | Cell lines | Up | PMC: 2907339, 20624269 |
| miR-326 | SCLC | Cell lines | Up | PMC: 2907339 |
| miR-324-5p | SCLC | Cell lines | Up | PMC: 2907339 |
