# Supplementary material for: A novel in silico reverse-transcriptomics-based identification and blood-based validation of a panel of sub-type specific biomarkers in lung cancer
Source: BMC Genomics. 2013 Oct 25;14(Suppl 6):S5. doi: 10.1186/1471-2164-14-S6-S5 (PMC3908344; doi:10.1186/1471-2164-14-S6-S5)
Supplement: Additional file 4 — Non-small-cell lung cancer (NSCLC) specific 143 deregulated miRNAs (89 upregulated and 43 downregulated). The miRNAs that are reported upregulated in one report but downregulated in other report or vise versa are highlighted in blue. [file 1471-2164-14-S6-S5-S4.doc]

**Additional file -4:** Non-small-cell lung cancer (NSCLC) specific 143 deregulated miRNAs (89 upregulated and 43 downregulated). miRNAs that are reported upregulated in one report but downregulated in other report or *vise versa* are highlighted in blue.

| **miRNAs** | **Type of cancer** | **Sample** | **Up/Down regulation** | **Exceptions** | **References** |
| --- | --- | --- | --- | --- | --- |
| let-7a-2 | NSCLC | Tissue | Down | Up in PMID: 16461460 | PMID: 16461460, PMID: 16530703 |
| let-7a | NSCLC | Tissue | Down | Up in PMID: 16461460 | PMID: 16461460,PMID: 18766170 |
| let-7g | NSCLC | Tissue | Down | Up in PMID: 16461460 | PMID: 16461460 |
| let-7f | NSCLC | Serum | Down |  | PMID: 18766170 |
| let-7g | NSCLC | Serum | Down |  | PMID: 18766170 |
| let-7i | NSCLC | Serum | Down |  | PMID: 18766170 |
| let-7e | NSCLC | Frozen lung SCC , Serum | Down |  | PMID: 19584273 ,PMID: 18766170 |
| let-7b | NSCLC | Serum | Up |  | PMID: 18766170 |
| let-7c | NSCLC | Serum | Up |  | PMID: 18766170 |
| let-7d | NSCLC | Serum | Up |  | PMID: 18766170 |
| mir-3 | NSCLC | Tissue | Down |  | PMID: 16530703 |
| miR-7-2 | NSCLC | Tissue | Up |  | PMID: 16461460 |
| miR-9-1 | NSCLC | Tissue | Up |  | PMID: 16461460 |
| miR-10a | NSCLC | Serum, tissue | Up |  | PMID: 18766170, PMID: 16461460 |
| miR-16-2 | NSCLC | Tissue | Up |  | PMID: 16461460 |
| miR-18b | NSCLC | Tissues | Up |  | PMID: 20818338 |
| miR-21 | NSCLC | Serum, sputum, tissue | Up |  | PMID: 19228723, PMID: 19273703 ,PMID: 18766170,PMID: 18719201, PMID: 21116241, PMID: 19493678, PMID: 16530703, PMID: 16461460 ,PMID: 20198613 |
| miR-26b | NSCLC | Serum | Down |  | PMID: 18766170 |
| miR-26a | NSCLC | Serum | Up |  | PMID: 18766170 |
| miR-27b | NSCLC | Serum | Up |  | PMID: 18766170 |
| miR-28-3p | NSCLC | Serum | Up |  | PMID: 18766170 |
| miR-28-5p | NSCLC | Serum | Up |  | PMID: 18766170 |
| miR-30a | NSCLC | Serum | Up |  | PMID: 18766170 |
| mir-30a-3p | NSCLC | Tissue | Down |  | NSCLC (Korean paper) |
| mir-30a-5p | NSCLC | Tissue | Down |  | PMID: 16461460 |
| mir-30c | NSCLC | Tissue | Down |  | NSCLC (Korean paper) |
| miR-30d | NSCLC | Serum/tissue | but Down in , NSCLC (Korean paper) | Up in PMID: 18766170 | PMID: 18766170, NSCLC (Korean paper),PMID: 16461460 |
| mir-30e-3p | NSCLC | Tissue | Down |  | NSCLC (Korean paper) |
| miR-32, | NSCLC | tissues. | Down |  | PMID: 20818338 |
| miR-34b, | NSCLC | Tissue | Up |  | PMID: 16461460 |
| miR-34c, | NSCLC |  | Down but | Up in PMID: 20818338 | PMID: 19228723, PMID: 20818338 |
| miR-92b | NSCLC | Serum | Up | Down in Korean Paper | PMID: 18766170 |
| miR-122 | NSCLC | Serum | Up |  | PMID: 18766170, |
| mir-124a-1 | NSCLC | Tissue | Up |  | PMID: 16530703, PMID: 16461460 |
| miR-125a-3p | NSCLC | NSCLC tissues | Down |  | PMID: 20569443 |
| miR-125a-5p | NSCLC | NSCLC tissues, Serum | Down |  | PMID: 20569443, PMID: 18766170 |
| miR-125a | NSCLC | Frozen lung SCC , Tissue | Down |  | PMID: 19584273 , NSCLC (Korean paper) |
| miR-125b | NSCLC | Serum, tissue | Up | Down in NSCLC (Korean paper) | PMID: 18766170, NSCLC (Korean paper) |
| miR-129-1/2 prec | NSCLC | Tissue | Up |  | PMID: 16461460 |
| miR-127 | NSCLC | Tissue | Up |  | PMID: 16461460 |
| miR-128a | NSCLC | Serum | Up |  | PMID: 18766170 |
| miR-130a | NSCLC | (NSCLC) tissues. | Up | Down in PMID: 18766170 | PMID: 20625274 |
| miR-132 | NSCLC | Tissue | Down |  | PMID: 16461460 |
| mir-133a-2 | NSCLC | Tissue | Down |  | NSCLC (Korean paper) |
| miR-134 | NSCLC | Serum | Up |  | PMID: 18766170 |
| miR-136 | NSCLC | Tissue | Up |  | PMID: 16461460 |
| miR-137 | NSCLC | tissues. | Down |  | PMID: 20818338, PMID: 18167339 |
| miR-139-3p | NSCLC | Serum | Up |  | PMID: 18766170 |
| miR-139-5p | NSCLC | Serum | Up |  | PMID: 18766170 |
| mir-140-5p | NSCLC | Tissue | Down |  | NSCLC (Korean paper) |
| miR-140-3p | NSCLC | Serum | Up |  | PMID: 18766170 |
| miR-141 | NSCLC | Tissue | Up |  | PMID: 16461460 |
| mir-144 | NSCLC | Tissue | Down |  | NSCLC (Korean paper) |
| miR-142-5p | NSCLC | Serum | Down |  | PMID: 18766170, PMID: 19228723 |
| miR-146a, | NSCLC | Serum | Up |  | PMID: 18766170 |
| miR-148 | NSCLC | Tissue | Up |  | PMID: 16461460 |
| miR-151-3p |  | Serum | Up |  | PMID: 18766170 |
| miR-151-5p |  | Serum | Up |  | PMID: 18766170 |
| miR-152 |  | Serum | Up |  | PMID: 18766170 |
| mir-181a |  | Tissue , Serum | Down | Up in PMID: 18766170 | NSCLC (Korean paper) |
| mir-181c |  | Tissue | Down |  | NSCLC (Korean paper) |
| miR-181d |  | Serum | Up |  | PMID: 18766170 |
| miR-181b |  | Serum | Up |  | PMID: 18766170 |
| miR-182, |  | lung cancer tissue , sputum | Up |  | PMID: 19493678, PMID: 19584273 , PMID: 20198613 ,PMID: 18167339 |
| miR-185 |  | Serum | Down |  | PMID: 18766170 |
| miR-186 |  | Serum | Up |  | PMID: 18766170 |
| mir-188 |  | Tissue | Down |  | PMID: 16530703 |
| miR-191 |  | Serum | Up |  | PMID: 18766170, PMID: 16461460 |
| miR-192 |  | Serum | Down |  | PMID: 18766170 |
| miR-193a-5p |  | Serum | Up |  | PMID: 18766170 |
| miR-195 |  | Tissue | Up |  | PMID: 16461460 |
| miR-196-1 |  | Tissue | Up |  | PMID: 16461460 |
| miR-196-2 |  | Tissue | Up |  | PMID: 16461460 |
| miR-197 |  | Serum | Upregulated |  | PMID: 18766170 |
| mir-198 |  | Tissue | Down |  | PMID: 16530703 |
| 199a-1 |  | Tissue | Up |  | PMID: 16461460 |
| miR-199a-2 |  | Tissue | Up |  | PMID: 16461460 |
| miR-199a-3p |  | Serum | Up |  | PMID: 18766170 |
| miR-199a-5p |  | Serum | Up |  | PMID: 18766170 |
| miR-199b |  | Tissue | Up |  | PMID: 16461460 |
| miR-199b-3p |  | Serum | Up |  | PMID: 18766170 |
| miR-200 |  |  | Down |  | PMID: 21115742 |
| miR-200b |  | Sputum/tissue | Up |  | PMID: 20198613 , PMID: 16461460 |
| miR-202 |  | Tissue | Up |  | PMID: 16461460 |
| mir-203 |  | Tissue | Up |  | PMID: 16530703 |
| mir-204-prec |  | Tissue | Down |  | PMID: 16530703 |
| miR-206 |  | Serum | Up |  | PMID: 18766170 |
| miR-210 |  | Sputum | Up |  | PMID: 20526284, PMID: 21116241 , PMID: 19493678, NSCLC (Korean paper), PMID: 16461460 |
| mir-212 |  | Tissue | Up |  | PMID: 16530703 |
| mir-214 |  | Tissue | Up |  | PMID: 16530703 |
| miR-215 |  | Tissue | Up |  | PMID: 16461460 |
| MicroRNA-218 |  | lung tissue | Down |  | PMID: 20838434 |
| mir-219-1 |  | Tissue | Down |  | PMID: 16530703 |
| mir-220 |  | Tissue | Down |  | PMID: 16530703 |
| mir-296-5p |  | Tissue | Down |  | NSCLC (Korean paper) |
| miR-320 |  | Serum | Up |  | PMID: 18766170 |
| miR-323-3p |  | Serum | Up |  | PMID: 18766170 |
| miR-330-3p |  | Serum | Up |  | PMID: 18766170 |
| miR-335 |  | Serum | Up |  | PMID: 18766170 |
| mir-338-3p |  | Tissue | Down |  | NSCLC (Korean paper) |
| miR-339-5p |  | Serum | Up |  | PMID: 18766170 |
| miR-339-3p |  | Serum | Up |  | PMID: 18766170 |
| miR-340 |  | Serum | Down |  | PMID: 18766170 |
| miR-342-3p |  | Serum | Up |  | PMID: 18766170 |
| miR-342-5p |  | Serum | Up |  | PMID: 18766170 |
| miR-342. |  | tissues. | Down |  | PMID: 20818338 |
| miR-345 |  | Serum | Up |  | PMID: 18766170 |
| miR-361-5p |  | Serum | Up |  | PMID: 18766170 |
| mir-371-3p |  | Tissue | Up |  | NSCLC (Korean paper) |
| miR-375 |  | Serum/sputum | Up |  | PMID: 18766170, PMID: 20198613 |
| mir-376b |  | Tissue | Down |  | NSCLC (Korean paper) |
| miR-378 |  | Serum | Down |  | PMID: 18766170 |
| miR-382 |  | Serum | upregulatd |  | PMID: 18766170 |
| miR-409-3p |  | Serum | Up |  | PMID: 18766170 |
| miR-409-5p |  | Serum | Up |  | PMID: 18766170 |
| miR-421 |  | Serum | upreguated |  | PMID: 18766170 |
| miR-423-5p |  | Serum | Up |  | PMID: 18766170 |
| miR-425 |  | Serum | Up | Down in NSCLC (Korean paper) | PMID: 18766170, NSCLC (Korean paper) |
| miR-432 |  | Serum | Up |  | PMID: 18766170 |
| miR-433 |  | Serum | Up |  | PMID: 18766170 |
| miR-451 |  | Serum | Down |  | PMID: 18766170 |
| miR-483-5p |  | Serum | Up |  | PMID: 18766170 |
| miR-485-3p |  | Serum | Up |  | PMID: 18766170 |
| miR-486, |  | Sputum | Down |  | PMID: 20198613 |
| miR-486-3p |  | Serum | Up |  | PMID: 18766170 |
| miRNA 486-5p |  | Plasma, Serum | Down |  | PMID: 21116241, PMID: 20194856 |
| miR-495 |  | Serum | Up |  | PMID: 18766170 |
| mir-497 |  | Tissue | Down |  | NSCLC (Korean paper) |
| miR-501-3p |  | Serum | Up |  | PMID: 18766170 |
| mir-520d-5p |  | Tissue | Down |  | NSCLC (Korean paper) |
| mir-520e |  | Tissue | Down |  | NSCLC (Korean paper) |
| mir-520f |  | Tissue | Down |  | NSCLC (Korean paper) |
| miR-543 |  | Serum | Up |  | PMID: 18766170 |
| miR-574-3p |  | Serum | Up |  | PMID: 18766170 |
| miR-574-5p |  | Serum | Up |  | PMID: 21258252 |
| miR-584 |  | Serum | Up |  | PMID: 18766170 |
| miR-589 |  | Serum | Up |  | PMID: 18766170 |
| miR-598 |  | Serum | Up |  | PMID: 18766170 |
| miR-629 |  | Serum | Up |  | PMID: 18766170 |
| miR-744 |  | Serum | Up |  | PMID: 18766170 |
| miR-766 |  | Serum | Up |  | PMID: 18766170 |
| miR-1254 |  | Serum | Up |  | PMID: 21258252 |
