# Supplementary material for: A novel in silico reverse-transcriptomics-based identification and blood-based validation of a panel of sub-type specific biomarkers in lung cancer
Source: BMC Genomics. 2013 Oct 25;14(Suppl 6):S5. doi: 10.1186/1471-2164-14-S6-S5 (PMC3908344; doi:10.1186/1471-2164-14-S6-S5)
Supplement: Additional file 5 — Functional annotation of common miRNAs using the targets of these miRNAs and DAVID [file 1471-2164-14-S6-S5-S5.doc]

**Additional file -5:** Functional annotation of common miRNAs using the targets of these miRNAs and DAVID.

| **miRNAs** | **Function Annotation** |
| --- | --- |
|  |  |
| miR-9 | **Transcription regulation** |
| miR-15a | **Regulation of cell proliferation**, Pathways in cancer |
| miR-16 | Protein Transport, Postive regulator of apoptosis, **Cell Division** |
| miR-17-5p | Focal Adhesion, Apoptosis, **Cell Death and Transcriptional Regulation** |
| miR-20a | **Proliferation**, Regulation in gene expression, apoptosis and p53 signaling pathway |
| miR-22 | Homeopoisis, apoptosis, **Regulation in transcription** |
| miR-23a | Positive regulation of phosphorylation, **regulation of cell motion** |
| miR-23b | Positive regulation of RNA metabolic process, **up-regulation of cellular biosynthetic process** |
| miR-25 | Response to steroid hormone stimulus, regulation of apoptosis |
| miR-24 | **Up-regulation of cellular biosynthetic process, regulation of apoptosis** |
| mir-27a | Regulation of apoptosis, **negative regulation of cellular biosynthetic process** |
| MicroRNA-29a | **Negative regulation of cellular biosynthetic process**, negative regulation of transcription |
| miR-29b | **Negative regulation of cellular biosynthetic process**, negative regulation of transcription |
| mir-29c | **Negative regulation of cellular biosynthetic process**, HOX |
| miR-34a | Regulation of phosphate metabolic process, regulation of apoptosis, regulation of kinase activity |
| miR-92a | Regulation of apoptosis, **negative regulation of cellular biosynthetic process**, positive regulation of nitrogen compound metabolic process |
| miR-93 | Negative regulation of gene expression, negative regulation of phosphate metabolic process |
| miR-99a | RNA splicing, via transesterification reactions, **negative regulation of cellular biosynthetic process**, organelle lumen |
| miR-101 | **Positive regulation of transcription**, cell projection morphogenesis |
| miR-103 | **Positive regulation of transcription, gene expression**, gonad development |
| miR-106a | Positive regulation of nitrogen compound metabolic process, Positive regulation of transcription, DNA-dependent, **Apoptosis** |
| miR- -106b | Positive regulation of nitrogen compound metabolic process, **Positive regulation of transcription,** **DNA-dependent, Apoptosis** |
| hsa-miR-107 | Membrane-enclosed lumen, Gene Silencing, **transcription regulation** |
| miR-126 | **Negative regulation of apoptosis**,regulation of phosphate metabolic process |
| miR-128b | RNA-mediated gene silencing, |
| miR-142-3p | **Positive regulation of cellular biosynthetic process**, negative regulation of transcription, DNA-dependent |
| miR-143 | Regulation of phosphorylation, regulation of kinase activity, **negative regulation of apoptosis** |
| miR-145 | **Negative regulation of apoptosis**, **positive regulation of cell migration,** regulation of phosphorylation |
| miR-149 | Positive regulation of nucleobase, nucleoside, nucleotide and nucleic acid metabolic process, **negative regulation of apoptosis,** positive regulation of cell migration |
| miR-150 | Positive regulation of transcription, DNA-dependent, protein amino acid phosphorylation, **positive regulation of apoptosis** |
| miR-155 | Positive regulation of apoptosis, protein import into nucleus, immune response-activating signal transduction |
| hsa-miR-183 | **Negative regulation of apoptosis, Negative regulation of cellular biosynthetic process and transcription** |
| miR-221 | Regulation of phosphorylation and protein kinase activity. Apoptosis. **Regulation of caspase activity** |
| miRNA-222 | Regulation of phosphorylation, **Positive regulation of apoptosis**, Positive regulation of phosphorylation |
| miRNA-205 | Positive regulation of nucleobase, nucleoside, nucleotide and nucleic acid metabolic process, **Transcription regulation, apoptosis** |
| miR-223 | Positive regulation of protein amino acid phosphorylation, hemopoietic or lymphoid organ development, **Positive regulation of cellular protein metabolic process** |
| mir-224 | Hemopoietic or lymphoid organ development, **Positive regulation of cell motion**, positive regulation of RNA metabolic process |
| miR-328 | Regulation of protein kinase activity, **regulation of apoptosis** |
