# Supplementary material for: A novel in silico reverse-transcriptomics-based identification and blood-based validation of a panel of sub-type specific biomarkers in lung cancer
Source: BMC Genomics. 2013 Oct 25;14(Suppl 6):S5. doi: 10.1186/1471-2164-14-S6-S5 (PMC3908344; doi:10.1186/1471-2164-14-S6-S5)
Supplement: Additional file 6 — Microarray based expression analysis of NSCLC specific 6 identified markers [E2F6, TFDP1, and SUV39H1 for NSCLC and RBL1, IRF1, and HMGA1 for general events]. E2F6, TFDP1, SUV39H1, and HMGA1 are significantly upregulated in both the adenocarcinoma and squamous cell carcinoma samples. The upregulation of RBL1 and downregulation of IRF1 in the microarray analysis was significant in squamous cell carcinoma but was statistically insignificant in adenocarcinoma. [file 1471-2164-14-S6-S5-S6.doc]

**Additional file-6:** Microarray based expression analysis of NSCLC specific 6 identified markers [E2F6, TFDP1, and SUV39H1 for NSCLC and RBL1, IRF1, and HMGA1 for general events]. E2F6, TFDP1, SUV39H1, and HMGA1 are significantly upregulated in both the adenocarcinoma and squamous cell carcinoma samples. The upregulation of RBL1 and downregulation of IRF1 in the microarray analysis was significant in squamous cell carcinoma but was statistically insignificant in adenocarcinoma.

|  |  |  | **Adenocarcinoma vs Normal** | | **Squamous carcinoma vs normal** | |
| --- | --- | --- | --- | --- | --- | --- |
| **GenBank** | **Affy Probe ID's** | **Gene Name** | **Fold Change** | **P-value** | **Fold Change** | **P-value** |
| NM_198256 | LC3P.9203C1.483a | **E2F6** | 1.76 | 0.011 | 2.26 | 0.000 |
| NM_198256 | LCMXR.7391C1_s |  | 1.69 | 0.015 | 2.35 | 0.000 |
| NM_198256 | LC3P.9203C1 |  | 1.53 | 0.021 | 2.98 | 0.000 |
| NM_198256 | LCMXR.7391C2 |  | 1.84 | 0.049 | 2.35 | 0.002 |
| NM_198256 | LC3SNG.2567a1_s |  | 1.06 | 0.788 | 1.20 | 0.395 |
| NM_007111 | LCRS.5543_s | **TFDP1** | 1.33 | 0.047 | 2.59 | 0.001 |
| AF550129 | LC3P.6768C1 |  | 1.33 | 0.072 | 2.65 | 0.002 |
| AF550129 | LCADNH.7414 |  | 1.52 | 0.181 | 2.15 | 0.009 |
| BF224431 | LCADNH.7617 |  | 1.26 | 0.367 | 1.47 | 0.007 |
| AF550129 | LCSS.8645 |  | -1.50 | 0.431 | 1.44 | 0.481 |
| AF550129 | LCADNH.8221 |  | -1.03 | 0.949 | -1.39 | 0.503 |
| NM_003173 | LC3P.9979C1 | **SUV39H1** | 2.07 | 0.016 | 2.22 | 0.004 |
| NM_003173 | LCMXR.15803C1 |  | 2.17 | 0.007 | 2.10 | 0.005 |
| NM_002895 | LCHP.402-22 | **RBL1** | 1.87 | 0.048 | 2.18 | 0.000 |
| NM_002895 | LCRS.10149 |  | 1.75 | 0.114 | 2.95 | 0.001 |
| NM_183404 | LCHP.402 |  | 2.03 | 0.146 | 2.21 | 0.000 |
| NM_002895 | LCAD.756 |  | 1.25 | 0.564 | 1.81 | 0.007 |
| NM_002895 | LCHPRC.402 |  | -1.13 | 0.723 | 1.09 | 0.000 |
| NM_002198 | LC3P.272C1 | **IRF1** | -1.69 | 0.149 | -2.22 | 0.004 |
| NM_002198 | LC3P.272C1.401a |  | -1.31 | 0.188 | -1.51 | 0.000 |
| NM_002198 | LCADA.15750 |  | -1.12 | 0.664 | -1.33 | 0.008 |
| NM_145901 | LCHP.941_s | **HMGA1** | 2.48 | 0.000 | 3.21 | 0.000 |
| NM_145901 | LCHP.325_s |  | 2.47 | 0.000 | 3.08 | 0.008 |
| NM_145901 | LCHP.941-22_s |  | 2.50 | 0.000 | 3.06 | 0.000 |
| NM_145901 | LC3P.810C1_s |  | 2.33 | 0.001 | 2.88 | 0.000 |
| NM_145901 | LCHP.1173 |  | 3.38 | 0.103 | 6.69 | 0.006 |
| L17131 | LCHPRC.945 |  | 1.62 | 0.106 | 3.82 | 0.007 |
| NM_145901 | LCHP.1173_x |  | 3.77 | 0.143 | 3.88 | 0.000 |
| NM_145901 | LCHPRC.1173_s |  | 2.51 | 0.162 | 3.04 | 0.002 |
| NM_145901 | LCHP.1173-22 |  | 1.51 | 0.190 | 4.28 | 0.000 |
| NM_145901 | LCHPRC.325_s |  | 4.24 | 0.197 | 3.87 | 0.175 |
